# Distribution of Endogenous Farnesyl Pyrophosphate and Four Species of Lysophosphatidic Acid in Rodent Brain

**DOI:** 10.3390/ijms11103965

**Published:** 2010-10-15

**Authors:** Sung Ha Lee, Siham Raboune, J. Michael Walker, Heather B. Bradshaw

**Affiliations:** 1 The Department of Psychological and Brain Sciences, Indiana University Bloomington, IN 47405, USA; E-Mails: sunghalee@gmail.com (S.H.L); sraboune@indiana.edu (S.R.); mtheodor@indiana.edu (J.M.W.); 2 The Gill Center for Biomolecular Science, Bloomington IN, 47405, USA; 3 The Kinsey Institute for Research in Sex, Gender and Reproduction, Bloomington IN 47405, USA

**Keywords:** LPA, FPP, GPR92, TRPV3, LC/MS/MS, pain

## Abstract

Lysophosphatidic acid (LPA) is the umbrella term for lipid signaling molecules that share structural homology and activate the family of LPA receptors. Farnesyl Pyrophosphate (FPP) is commonly known as an intermediate in the synthesis of steroid hormones; however, its function as a signaling lipid is beginning to be explored. FPP was recently shown to an activator of the G-protein coupled receptor 92 (also known as LPA5) of the calcium channel TRPV_3_. The LPA receptors (including GPR92) are associated with the signal transduction of noxious stimuli, however, very little is known about the distribution of their signaling ligands (LPAs and FPP) in the brain. Here, using HPLC/MS/MS, we developed extraction and analytical methods for measuring levels of FPP and 4 species of LPA (palmitoyl, stearoyl, oleoyl and arachidonoyl-sn-glycerol-3 phosphate) in rodent brain. Relative distributions of each of the five compounds was significantly different across the brain suggesting divergent functionality for each as signaling molecules based on where and how much of each is being produced. Brainstem, midbrain, and thalamus contained the highest levels measured for each compound, though none in the same ratios while relatively small amounts were produced in cortex and cerebellum. These data provide a framework for investigations into functional relationships of these lipid ligands in specific brain areas, many of which are associated with the perception of pain.

## 1. Introduction

Lysophosphatidic acid (LPA) is not a single molecule, but a collection of signaling molecules that share structural homology and activate the family of LPA receptors. LPAs are structurally composed of a fatty acid linked to *sn*-glycerol-3 phosphate (e.g., arachidonoyl-*sn*-glycerol-3 phosphate). They induce their effects through binding to members of the superfamily of G protein-coupled receptors (GPCRs) including LPA1 (also named VZG-1, MREC1.3 or EDG-2) [1,2], LPA2 (or EDG-4) [3], LPA3 (or EDG-7) [4,5], LPA4 (formerly GPR23) [6] and more recently discovered LPA5 (formerly GPR92) [7]. These receptors couple to at least three different types of G proteins, notably G_q_/_11_, G_i/o_ and G_12/13_ [8]. LPA receptors coupled to G_i_ proteins drive the activation of the PI3K-Akt pathway [9], whereas, LPA receptors bound to G_12/13_ activate the small GTPase RhoA [10]. LPA ligands have been known to influence a wide range of cellular functions including cell proliferation, apoptosis, and migration [9,11,12]. Overwhelming evidence points to the function of LPA in pain because LPA (1) stimulates cyclooxygenase-2 expression (COX-2) [13], which contributes to inflammatory pain; (2) increases reactive oxygen species (ROS) release [14], which is involved in the development and maintenance of neuropathic pain [15]; (3) induces nociception-producing activity and substance P release on sensory neurons [16]; and (4) is involved in the initiation of neuropathic pain [3,17,18]

Farnesyl pyrophosphate (FPP) is a key intermediate in the biosynthesis of steroids including cholesterol as well as the donor of the farnesyl group for isoprenylation of many proteins, including the βγ subunit of some G proteins and GTPases, Ras and Rho [19]. FPP is synthesized from farnesol via two successive phosphorylation reactions mediated by farnesol kinase and farnesyl phosphate kinase, transitioning first through farnesyl monophosphate (FMP) then to FPP [20].

The actions of a class of cholesterol-lowering drugs called Statins (e.g., Atorvastatin, Lipitor) suggest the impact of FPP on pain. These drugs inhibit HMG-CoA reductase (3-hydroxsyl-3- methylglutaryl coenzyme A reductase), the first committed enzyme of the cholesterol synthesis pathway [21]. Recent data indicate that Statins are also anti-inflammatory and anti-nociceptive compounds [22]. This report also showed that Atorvastatin lowers hyperalgesia produced by bradykinin and cytokines as well as being associated with a drop in inflammatory mediators such as TNF-α and IL-1β. Because FPP is a key intermediate for cholesterol synthesis, a reduced level of such intermediates may contribute to reduced hyperalgesia.

Recent data indicated that FPP acts as a ligand of GPR92/LPA_5_ [23], suggesting an underlying mechanism for FPP in relation to pain. Activation of GPR92/LPA_5_ by FPP increases IP production, cAMP levels, and Ca^2+^ levels in a concentration-dependent manner. Oh and colleagues further demonstrated that FPP and LPA are able to activate G_q/11_ and G_s_-mediate signaling pathways. Evidence for a role in pain comes from the fact that GPCR92/LPA_5_ is highly expressed in and largely co-localized with TRPV1 in mouse and human dorsal root ganglion [23], suggesting that this receptor may contribute to the processing of noxious sensory stimuli. However, FPP may not interact with GPR92 in a typical receptor-ligand manner. A report by Yin and colleagues [24] states that FPP was not effective in recruiting beta arrestin in a GPR92 expression system. A more recent report shows that FPP is also a potent activator of TPRV_3_, which adds yet another dimension on how FPP may play a role in the processing of noxious stimuli [25]. It will be important to investigate any potential interactions of GPR92/LPA5 with TRPV3 to further elucidate this complex signaling system.

In order to begin investigations of FPP and LPA’s functional relevance as signaling molecules in the central nervous system, we developed HPLC/MS/MS extraction protocols for each compound from brain tissue and examined the relative distribution of FPP and four different endogenous LPAs; oleoyl-LPA (O-LPA), arachidonyl-LPA (A-LPA), palmitoyl-LPA (P-LPA) and stearoyl-LPA (S-LPA) were measured here in 9 distinct regions of the brain. Our findings show there is not a uniform distribution throughout the brain of any of the 5 compounds measured lending support to the hypothesis that they are working as signaling molecules and not simply as structural lipids. While the relative distributions are not the same, the highest concentrations of each are found primarily in brainstem and midbrain providing a focus for future research.

## 2. Results and Discussion

### 2.1. Distribution of FPP in Rat Brain

**Figure 1:**
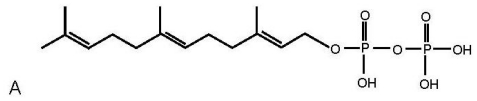

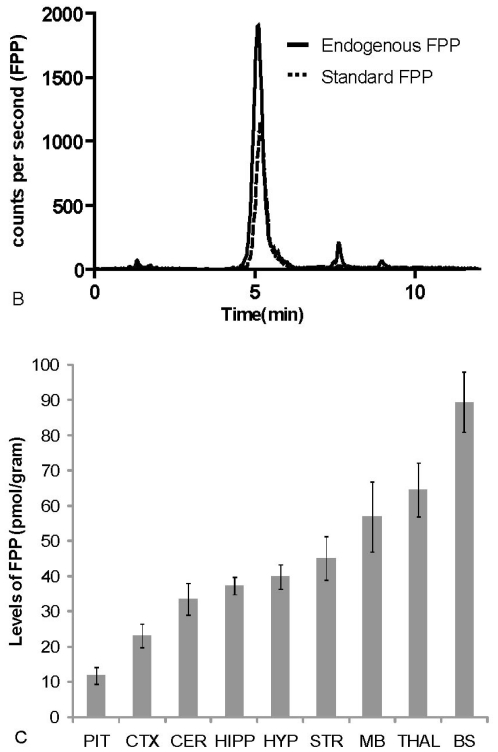
(**A**) structure of FPP (**B**) co-eluting peaks for synthesized FPP (standard FPP) and a representative brain constituent (Endogenous FPP) (**C**) distribution of FPP in selective brain areas. Abbreviations: PIT, pituitary; CTX, cortex; CER, cerebellum; HIPP, hippocampus; HYP, hypothalamus; STR, striatum; MB, midbrain; THAL, thalamus; BS, brain stem.

Co-eluting peaks for synthesized FPP and a brain constituent that matched the HPLC/MS/MS fingerprint of FPP were observed in each area of the brain examined (representative brain region shown in Figure 1). The average rate of recoveries across brain areas was 59%. FPP was not distributed equally throughout the brain (Figure 1). The highest production of FPP was measured in the brainstem (89.55E-12 ± 17.10E-12 moles/gram tissue), followed by the midbrain (56.95E-12 ± 19.89E-12) and thalamus (64.95E-12 ± 15.20E-12), moderate levels were measured in the hypothalamus (39.91E-12 ± 6.8E-12), cerebellum (33.62E-12 ± 8.94E-12), hippocampus (37.31E-12 ± 4.98E-12) and striatum (45.19E-12 ± 12.51E-12), and the lowest levels were measured in the pituitary (11.81E-12 ± 4.81E-12) and cortex (23.09E-12 ± 6.78E-12).

### 2.2. Distribution of Four LPA Species in Rat Brain

**Figure 2:**
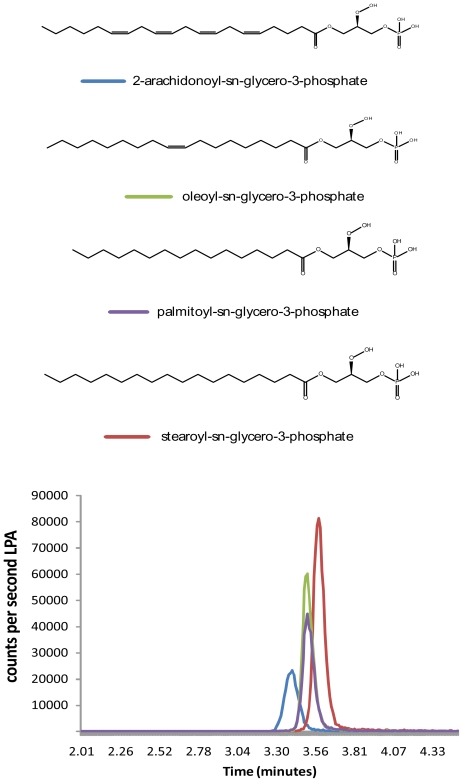
Structure of four LPA species and corresponding HPLC/MS/MS chromatographs for each of these species from brain extracts. Colors for each chromatographic peak correspond to the color bar next to the name of the LPA. Using the C18 reversed phase analytical column the least saturated LPA (arachidonoyl-sn-glycerol-3 phosphate) elutes first and the longest and completely saturated LPA (stearoyl-sn-glycerol-3 phosphate) elutes last.

Co-eluting peaks for all four synthesized LPA molecules and a corresponding brain constituent were observed in each area of the brain examined. Figure 2 demonstrates the chromatograms of the LPAs HPLC/MS/MS methods in which each LPA species is measured from the complex brain lipid extract. Using the C18 reversed-phased analytical column the arachidonoyl species elutes first, followed by the palmitoyl, oleoyl and finally stearoyl species.

Like FPP, the highest amounts of LPAs were measured in the brainstem, midbrain, and thalamus, though not equally distributed (Table 1). Relative abundance of each LPA species was not consistent in any brain areas tested (Table 1). A-LPA shows the most divergence from the other three LPA species in that it is least abundant in cortex and the levels in the hypothalamus are equivalent to those in the brainstem. Averaged recoveries were equivalent across compounds: A-LPA (90 ± 6%), O-LPA (70 ± 17%), P-LPA (70 ± 23%), and S-LPA (65 ± 8%).

### 2.3. Discussion

This study provides a framework to examine FPP and LPA production that will direct future studies on functional relevance of each as signaling molecules. This is particularly important in that these data show that the relative distributions are significantly different and, therefore, their contributions as substrates and signaling molecules is dependent on the population and phenotype of cells within a region. That there is not an equal distribution throughout the brain also provides evidence to suggest that the use of plasma levels of these lipids is an inadequate method for measuring central nervous system activity. While these lipids are detectable in plasma, it is important to recognize the distinction between those derived likely from circulating immune cells and those that are made on demand within a specific region of the CNS.

The highest levels of FPP, A-LPA, O-LPA and P-LPA were detected in the brain stem and midbrain in our study. Interestingly, these areas overlap with the descending modulatory system for pain. Rostral ventromedial medullar (RVM) in the brainstem was established as an integral relay in the descending modulation of pain [26,27]. Additionally, the periaqueductal gray (PAG) has extensive involvement in the descending inhibition of pain and is a rich area of study for both opioid [26] and cannabinoid analgesia [28,29].

Electrical stimulation in the lateral and medial midbrain PAG and intraplantar formalin injection caused significant increases in the production of the endogenous cannabinoid anandamide in the PAG [29]. Acute stress was also shown to suppress pain by increasing the production of the endocannabinoids 2-AG and anandamide in the PAG region, which are known to be associated with stress-induced analgesia [28]. In the same study there was a relationship with the elevation of endocannabinoid levels in the PAG after injection of inhibitors of endocannabinoid degradation enzymes and an increase in tolerance to noxious stimuli. Here we show that FPP and LPA are significantly more abundant in the midbrain and brainstem. Their abundance here may be an indication that they are involved in the descending regulatory pathways for pain including the PAG and the RVM, providing yet another aspect of the fine-tuning of the PAG and RVM systems.

By examining the relative levels of these lipids in distinct brain regions we also demonstrate that they are not merely structural components of the cell membrane. If they were simply produced in this capacity their levels should be more consistent throughout the brain. Even with the growing interest in LPAs and FPP as signaling molecules over the last decade, few studies have examined levels of these lipids in the CNS. One study measured the level of FPP using LC/MS/MS in human frontal cortex tissue [30] from post-mortem brains in five elderly patients and found it to be in the range of 4.5 ng/mg. Here, we show that levels of FPP in rat cortex are ~7 ng/mg, so these levels are in an equivalent range.

A current therapy aimed at the reduction of lipogensis related to high blood pressure and heart disease is the use of Statins. These are already known to reduce hypernociception likely mediated by changes in the levels of bradykinin and cytokines, IL-1β, and PGE_2_ [22]. However, the antinociceptive effect of Statins also appears to be tightly related to reducing the production of FPP in that exogenous malvonate, the precursor of FPP, reversed the antinociception by Statins in the same study. Also, inhibition of isoprenoid production by Statins has been shown to increase NOS expression which prevents hypernociceptive activity [31]. Although Statins do not cross the blood brain barrier [32], the peripheral action of statins may reduce central sensitization by decreasing peripheral nociceptive input to the central nervous system [33]. Along with HMG CoA reductase, bisphosphonate is also known to decrease FPP levels primarily by inhibiting farnesyl diphosphate synthase (FPPS) [34]. Interestingly, bisphosphanate also has antinociceptive effects [35] in that chronic treatment with bisphosphanate attenuated hyperalgesia associated with chronic peripheral neuropathy and inflammation.

The roles of LPAs in pain are also currently under intense study and have been recently reviewed by many authors [36,37]. LPA causes reactive oxygen species (ROS) release [38], which is involved in signaling of noxious stimuli and the perception of pain [39,40]. LPA-induced activation of phosphatidylinositol–3 kinase (PI3K) has been suggested to play an essential role in the induction of pain hypersensitivity [41]. PI3K is involved in capsaicin-induced heat hyperalgesia, perhaps through TRPV1 sensitization in that capsaicin-induced TRPV1 current enhancement was blocked by PI3K inhibition. LPA was also shown to cause sensory neuron sensitization through G_i/0_ activation and substance P (SP) release [42]. Substance P directly activates IL-8 release from macrophages leading to hyperalgesia through sympathetic neurons stimulation [43,44]. Moreover, Inoue M. et *al*. showed that intrathecal injection of LPA into wild-type animals elicited mechanical allodynia and thermal hyperalgesia effects similar to those induced by peripheral nerve injury [18]. These effects of LPAs on peripheral sensory processing underscore their potency and the need to understand LPA functions in the CNS.

## 3. Experimental Section

### 3.1. Sample Preparation

Hypothalamus, pituitary, thalamus, brainstem, cerebellum, hippocampus, striatum, cortex and midbrain were dissected from 10 female Sprague-Dawley rats and immediately frozen at −80 °C until used for extractions. For FPP extractions, tissue was homogenized on ice in an ethanol/butanol/70 mM (1:1.25:1.25. v/v/v) ammonium hydroxide solution using a polytron homogenizer. The homogenate was centrifuged at 20,000 g, 25 °C for 5 min. The supernatant was loaded on polymeric solid phase extraction columns (Strata-X SPE, Phenomena), washed with 40% HPLC methanol and eluted with 100% HPLC methanol/1% triethyl amine. For LPA extractions, tissue was homogenized in 100% HPLC-grade methanol, homogenate was centrifuged at 20,000 g, 25 °C for 20 min, the supernatant then diluted with HPLC-grade water to make a 30% organic solution which was then loaded on pre-conditioned Empore C8 SPE columns (Varian, Palo Alto, CA), columns were washed with HPLC-grade water, 40% HPLC-grade methanol, and eluted with 100% HPLC-grade methanol.

### 3.2. HPLC/MS/MS

Levels of each compound were analyzed by multiple reactions monitoring (MRM) mode on a triple quadrupole mass spectrometer API 3000, with electrospray ionization. Samples were loaded using Shimadzu SCL10Avp autosampler. The flow rate was 200 uL/min achieved by a system comprised of a Shimadzu controller and two Shimadzu LC10ADvp pumps.

#### 3.2.1. FPP Analysis

The chromatographic analysis was performed on a C18 reversed phase column (Phenomenex Gemini C18 column, 50 × 2.0 mm, 3 μM) guarded by SecurityGuard Cartridge System (Phenomenex). Mobile phase A, 1.0% triethyl amine (TEA), and mobile phase B, 90% isopropanol with 1.0% TEA, were used for gradient elution. Mobile phase B was held constant at 2% for 2 min (mobile phase A at 98%) after sample injection, then a linear gradient increased B to 100% between 2 and 12 min, which was maintained for 2 min and brought back to 2% at 14.50 prior to 2 minutes of column equilibration. Columns were maintained at 40 °C. Recoveries were determined by spiking supernatant from paired brain areas and analyzing the% recovery of the spiked samples. FPP levels were determined using MRM in negative ion mode where the parent mass (MH-) was 381.0 m/z and the two product ions measured where 79.0 and 158.8 m/z.

#### 3.2.2. LPA Analysis

The chromatographic analysis was performed on a 210 mm Zorbax Eclipse XDB-C18 reversed phase HPLC (3.5 um internal diameter) column maintained at 40 °C. Mobile phase A: 80/20 H_2_O/MeOH with 10mM ammonium acetate and 1% acetic acid and mobile phase B: 100% Methanol with 10mM ammonium acetate and 1% acetic acid were used for gradient loading and elution. LPA parent and fragment masses monitored via MRM are as follows: P-LPA parent mass 409.48 fragment mass 153.10; O-LPA parent mass 435.57 fragment mass 153.10; S-LPA parent mass 437.27 fragment mass 153.10; and A-LPA parent mass 457.30 fragment mass 153.10. Due to a lack of deuterium-labeled standards to use as internal standards to calculate percent recoveries, they were determined by using the alternate half of the brain dissections from those used for extractions of unknown amounts and spiking brain areas with μM quantities of each LPA subtype to estimate overall recoveries.

## 4. Conclusions

In this study, levels of FPP and four species of LPA were shown to be differentially produced throughout the rodent brain. These data provide a framework for investigations into the functional relationships of these lipid ligands in these specific brain areas, many of which are associated with pain.

## Figures and Tables

**Table 1:** Levels of 4 LPA species in 8 areas of the rat brain determined by HPLC/MS/MS. Amounts are listed in acceding order by brain area.

P-LPA in nmols/gr		A-LPA in pmols/gr	
Hypothalamus	4.22 ± 0.81	Cortex	220 ± 30.2
Hippocampus	6.16 ± 0.37	Cerebellum	285 ± 25.1
Cortex	6.96 ± 1.19	Striatum	299 ± 23.2
Striatum	7.88 ± 1.11	Hippocampus	316 ± 15.3
Cerebellum	13.4 ± 1.58	Hypothalamus	339 ± 67.6
Thalamus	19.6 ± 0.58	Brain Stem	364 ± 16.3
Brain Stem	25 ± 3.17	Thalamus	478 ± 29.6
Midbrain	28.6 ± 2.26	Midbrain	536 ± 41.8
**S-LPA in nmols/gr**		**O-LPA in nmols/gr**	
Hypothalamus	4.34 ± 1.64	Hypothalamus	7.58 ± 1.83
Hippocampus	6.30 ± 0.62	Hippocampus	9.76 ± 1.22
Striatum	7.68 ± 0.46	Striatum	11.4 ± 1.01
Cortex	10.7 ± 0.55	Cortex	17.5 ± 3.27
Cerebellum	11.2 ± 0.51	Thalamus	37.8 ± 4.05
Brain Stem	13 ± 0.56	Cerebellum	28.1 ± 5.34
Midbrain	14 ± 1.99	Midbrain	63.6 ± 7.48
Thalamus	15.7 ± 2.24	Brain Stem	69.5 ± 4.30
